# Characterization of miRNA profiling in konjac-derived exosome-like nanoparticles and elucidation of their multifaceted roles in human health

**DOI:** 10.3389/fpls.2024.1444683

**Published:** 2024-08-08

**Authors:** Chuan Shen, Xia Li, Jianfeng Qin, Longfei Duan

**Affiliations:** ^1^ Shaannan Eco-economy Research Center, Ankang University, Ankang, China; ^2^ Department of Electronic and Information Engineering, Ankang University, Ankang, China; ^3^ Ankang Municipality Agricultural Science Research Institute, Ankang, China

**Keywords:** konjac, ultracentrifugation, exosome-like nanoparticles, miRNA profiling, human disease

## Abstract

Plant-derived exosome-like nanoparticles (ELNs) have demonstrated cross-kingdom capabilities in regulating intercellular communication, facilitating drug delivery, and providing therapeutic interventions in humans. However, the functional attributes of konjac-derived ELNs (K-ELNs) remain largely unexplored. This study investigates the isolation, characterization, and functional analysis of K-ELNs, along with the profiling and differential expression analysis of associated miRNAs in both K-ELNs and Konjac tissues. K-ELNs were successfully isolated and characterized from two konjac species using ultracentrifugation, followed by Transmission Electron Microscopy (TEM) and Nanoparticle Tracking Analysis (NTA). Small RNA sequencing identified a total of 3,259 miRNAs across all samples. Differential expression analysis revealed significant differences in miRNA profiles between K-ELNs and tissue samples. Gene Ontology (GO) and Kyoto Encyclopedia of Genes and Genomes (KEGG) functional enrichment analysis of target genes provided insights into their roles in modulating pathways associated with diseases such as cancer and neurodegenerative disorders. Additionally, six miRNAs were selected for validation of sequencing results via RT-qPCR. The 5’RLM-RACE method was employed to validate the cleavage sites between differentially expressed miRNAs (DEMs) and their predicted target genes, further substantiating the regulatory roles of miRNAs in konjac. The findings of this study enhance our understanding of the molecular mechanisms underlying the biological functions and applications of K-ELNs, laying the groundwork for future research into their potential therapeutic roles in human health.

## 1 Introduction

Plant-derived exosome-like nanoparticles (ELNs), ranging in size from 50 to 200 nm, have recently garnered attention due to their significant implications in intercellular communication and biomedical applications. Similar to mammalian exosomes, these nanoscale vesicles are released by various plant cells and contain a diverse cargo of biomolecules, including proteins, lipids, nucleic acids, and small molecules (Kim et al., 2022). ELNs play pivotal roles in mediating cell-to-cell communication, enabling the exchange of information and molecular signals among different cells and tissues within plants (Suharta et al., 2021). Among their key functions, ELNs are instrumental in modulating various physiological processes in plants, including growth, development, and responses to stress. By acting as carriers of signaling molecules such as hormones and defense-related compounds, ELNs facilitate the orchestration of plant responses to environmental cues and biotic stresses (Zhang et al., 2022). Moreover, ELNs have been implicated in mediating interactions between plants and microbes, promoting symbiotic associations and bolstering defense mechanisms against pathogens (Rutter and Innes, 2018).

In addition to their roles in plant biology, ELNs hold promise for diverse applications in biomedicine, particularly in the realms of drug delivery and therapeutics. Due to their inherent biocompatibility and ability to traverse biological barriers such as cell membranes and the blood-brain barrier, ELNs present opportunities as carriers for targeted drug delivery and gene therapy (Sarasati et al., 2023). For instance, ELNs derived from ginseng have been shown to influence macrophage polarization, resulting in the suppression of melanoma growth (Cao et al., 2019). Similarly, ELNs sourced from ginger can modulate the composition of specific bacterial species and impact microbial metabolism, thereby influencing host health and disease outcomes (Teng et al., 2018). In the realm of inflammatory treatment, broccoli-derived ELNs activate dendritic cell AMP-activated protein kinase (AMPK), leading to anti-inflammatory effects in colitis (Deng et al., 2017). Comparable therapeutic effects have been observed with grape-derived ELNs and garlic chives-derived ELNs in primary macrophages (Ju et al., 2013; Liu et al., 2021). Furthermore, citrus-derived ELNs exert regulatory effects on inflammatory gene expression and the maintenance of tight junctions in the intestinal epithelium (Bruno et al., 2021). In the domain of drug delivery, lemon-derived ELNs have shown promise as a natural and potentially effective therapeutic strategy for gastric cancer (Yang et al., 2020). Additionally, strawberry-derived ELNs have demonstrated the ability to mitigate oxidative stress in human mesenchymal stromal cells in a dose-dependent manner (Perut et al., 2021).

MicroRNAs (miRNAs) represent a class of small noncoding RNAs that play crucial roles in physiological and pathological processes by modulating the expression of target genes. Until a decade ago, the unique biological effects of plants were mainly attributed to their phytochemical content. However, recent studies have highlighted the beneficial properties of medicinal plants associated with their miRNA content (Lukasik et al., 2018; Avsar et al., 2020). This emerging understanding has the potential to change perspectives on the role of plant foods and medicinal plants (Gismondi et al., 2021). A plethora of exosome-secreted miRNAs have been identified from various edible plants, underscoring their significance in intercellular communication and molecular regulation spanning different biological kingdoms (Xiao et al., 2018). Encapsulated within plant-derived ELNs, these small non-coding RNAs fulfill diverse functions in orchestrating physiological processes and governing gene expression in recipient cells (Yin et al., 2023). Distinguishing themselves from conventional signaling molecules, miRNAs carried by ELNs possess the ability to traverse cellular barriers and influence gene expression in target cells, thereby showcasing their potential as novel regulators of biological pathways (Leng et al., 2024). For instance, ELNs derived from citrus have demonstrated substantial inhibition of Penicillium italicum mycelium growth on citrus fruit (Yin et al., 2023). Furthermore, miRNAs conveyed by ELNs exhibit remarkable capabilities in cross-kingdom regulation, impacting gene expression in non-plant organisms such as animals and humans. Increasing evidence suggests that plant-derived miRNAs can be internalized by mammalian cells, where they modulate gene expression and biological processes, thereby offering promise as therapeutic agents and diagnostic biomarkers (Leng et al., 2024). For instance, ELNs from maize selectively target endogenous porcine mRNAs upon entry into the gastrointestinal tract, exerting effects on gene expression akin to mammalian miRNAs (Luo et al., 2017). Additionally, plant miR168a has been shown to target human/mouse LDLRAP1 mRNA, leading to the inhibition of its expression in the liver (Zhang et al., 2011). Similarly, plant miR159 has demonstrated the ability to suppress the proliferation of breast cancer cells and impede the growth of breast tumors by targeting the transcription factor TCF7 (Chin et al., 2016).

Research on plant ELNs and their associated miRNAs has provided valuable insights into their biogenesis, composition, and biological functions. The characterization of plant ELNs and their miRNA cargo represents a rapidly advancing field, with advancements in analytical techniques facilitating comprehensive exploration of their biophysical properties and molecular profiles. Various methods are employed for the isolation of exosomes, including differential centrifugation, density gradient centrifugation, volumetric exclusion chromatography, filtration, polymer precipitation, immunoseparation, and isolation screening. Among these methods, ultracentrifugation stands as the predominant separation technique in current exosome research, enabling the precise and reproducible isolation of exosomes while minimizing co-purification with protein aggregates and other membrane particles. Studies have leveraged techniques such as ultracentrifugation and RNA sequencing to unveil the composition and functional diversity of plant ELNs (Yi et al., 2023). Furthermore, bioinformatics tools have been harnessed to predict the target genes of plant-derived miRNAs, offering insights into their regulatory networks. In a previous study, high-throughput small RNA sequencing was employed to isolate and identify miRNAs from coconut water ELNs (Zhao et al., 2018).

*Amorphophallus konjac* K. Koch, commonly referred to as konjac, is a perennial herbaceous tuberous plant belonging to the genus *Amorphophallus Blume* within the family *Araceae*. It is extensively cultivated in the mountainous regions of China, Japan, and Southeast Asia (Ulrich et al., 2016). Konjac is recognized as a natural health food and a source of medicinal raw materials. The bulbs of konjac are notably abundant in glucomannan (KGM), which holds significant value in processing and health promotion (Nishinari et al., 1992). For instance, KGM can enhance gastrointestinal motility, facilitate protein absorption, and effectively regulate abnormal increases in human cholesterol and blood lipids. Moreover, it contributes to improving the human immune system and offers preventive and adjunct therapeutic effects for cardiovascular diseases, obesity, and digestive tract cancers (Chua et al., 2010; Behera and Ray, 2016). Despite its diverse benefits, research on the molecular biology of konjac has commenced relatively later compared to other plant species.

In this study, exosome-like nanoparticles derived from konjac (K-ELNs) were isolated, and small RNA sequencing was subsequently employed to identify differentially expressed miRNAs (DEMs) between exosomes and tissues from two konjac varieties. This research represents the first comprehensive characterization of the morphology and dimensions of konjac exosomes. Furthermore, we identified a series of both known and novel miRNAs and conducted functional annotations. The primary objective of this study is to provide an in-depth characterization of K-ELNs and their associated miRNAs, with particular emphasis on investigating their potential human gene targets across various biological kingdoms. Our goal is to elucidate the regulatory mechanisms underlying K-ELN-mediated intercellular communication and to explore their implications for human health and disease.

## 2 Materials and methods

### 2.1 Pretreatment of konjac

The konjac samples were sourced from Ankang Academy of Agricultural Science, Shaanxi, China (32°41’N, 109°01’E, altitude 800 m). Two species of konjac, namely *Amorphophallus konjac* (*A. konjac*) and *Amorphophallus albus (A. albus)*, designated as HM and BM, were utilized in the experimental procedures of this study. Samples were collected after the ‘changing head’ stage. For each variety, we selected healthy plants with similar growth characteristics. Approximately 200 g of konjac tubers were meticulously cleaned and grated with phosphate-buffered saline (PBS) (E607008, Sangon Biotech) for each sample. Subsequently, the mixture underwent a series of centrifugation steps to eliminate large particles and other impurities. All processing steps were carried out at 4°C.

### 2.2 Isolation of K-ELNs by ultracentrifugation

The extracellular vesicles (EVs) from konjac samples were isolated through a combination of high-speed and ultracentrifugation (CP100MX, Hitachi, Japan) at 100,000 rpm for 2 hours. The obtained pellet was re-suspended in distilled water, further resuspended in PBS, and stored at -80°C for subsequent analysis. The EVs obtained from HM and BM samples were designated as HM_Exo and BM_Exo, respectively.

### 2.3 Transmission electron microscopy

A volume of 10 μL of the EV sample was applied to a copper grid for 1 minute, after which any excess liquid was removed using filter paper. Subsequently, 10 μL of dioxiranyl acetate (GZ02625, Zhongjingkeyi Technology Co., Ltd, China) was added to the copper grid for 1 minute and allowed to dry at room temperature for several minutes, with excess liquid removed using filter paper. The morphologies and distribution states of the EVs were examined using transmission electron microscopy (HT-7700, Hitachi, Japan) at an acceleration voltage of 100 kV.

### 2.4 Nanoparticle tracking analysis

The frozen EV samples were thawed in a water bath at 25°C and then immediately placed on ice. Subsequently, the samples were re-suspended and diluted with PBS. The particle size distribution of the EVs was analyzed using a nanoparticle tracking analyzer (ZetaVIEW, Particle Metrix, Germany).

### 2.5 RNA extraction and small RNA sequencing

The RNA extraction from tissue and EV samples was conducted using a plant total RNA extraction kit (DP441, Qiagen). Following extraction, 1 μL of each RNA sample was reserved for staining, and the RNA concentration was quantified using a Quantus Fluorometer (Promega, USA). The EV RNA stock solution was diluted with NR1 Card Clip Matching Diluent and subsequently analyzed using the bio-fragment analyzer Qsep100 (BIOptic, Taiwan).

The miRNA extraction was performed using a miRNeasy Mini kit (217004, Qiagen). The miRNA extraction process involved sample homogenization in a denaturing buffer, phase separation with chloroform, and collection of the aqueous phase. This was mixed with ethanol and processed through an RNeasy Mini column. After centrifugation and washing to remove impurities, purified miRNA was eluted with RNase-free water. Subsequently, twelve small RNA libraries were prepared through the sequential steps of 3’ adaptor ligation, 5’ adaptor ligation, reverse transcription with unique molecular index (UMI) assignment, cDNA cleanup, library amplification, and sample index assignment. The constructed libraries were then sequenced on the Illumina NextSeq PE150 platform, and the quality of the sequenced data was evaluated using FastQC. The construction and sequencing of the libraries were facilitated by GeneCreate (Wuhan, China).

### 2.6 Identification of miRNA and DEMs

Clean reads were acquired from the raw reads through the elimination of N bases at both ends of the sequence, Q20 filtration, and adaptor excision utilizing the FASTAX-Toolkit. The clean reads (18- to 30-nt) were aligned to *Amorphophallus konjac* draft genome (PRJNA734512) and then mapped to Rfam (https://rfam.xfam.org/) using the Bowtie short sequence comparison tool to remove ncRNAs such as rRNAs, tRNAs, snRNA, and snoRNA (Langmead et al., 2009). The remaining clean reads were mapped to the miRBase database (release 22, http://www.miRBase.org/) to identify known miRNAs (Griffiths-Jones, 2006). The discovery of novel miRNAs and prediction of secondary structures were conducted using the RNAfold and miRDeep2 software tools (Friedländer et al., 2011). However, it is important to note that our analysis of *A. albus* sequencing data was conducted using the *A. konjac* genome as a reference, due to the current unavailability of a published *A. albus* genome.

The abundance of identified miRNAs in the various samples was normalized to transcripts per kilobase million (TPM). The R-based DESeq2 package was employed to ascertain the variance in expression patterns of the identified miRNAs between EVs and tissues. An miRNA was deemed significantly differentially expressed if it demonstrated a log 2 (fold change) ≥1 or ≤ −1 with a significance level of p-value < 0.05.

### 2.7 Prediction and functional annotation of miRNA target genes

The prediction of miRNA-mediated target genes was carried out using the miRanda software with default parameters (sc 140, en -7.0) (Enright et al., 2003). Konjac miRNA sequences were selected as query sequences, and both the konjac draft genome and human genome were utilized as the reference databases. Potential targets were determined based on BLASTn hits with fewer than three mismatches. Subsequently, gene ontology (GO) and Kyoto Encyclopedia of Genes and Genome (KEGG) pathway enrichment analysis, as well as functional annotation of DEMs target genes, were performed using the ClusterProfiler (v4.1.4) R package, with a cutoff of adjusted P-adj < 0.05 (Wu et al., 2021).

### 2.8 Quantitative real-time PCR

The validation of expression levels for 6 DEMs, including 4 up-regulated miRNAs and 2 down-regulated miRNAs were carried out using stem-loop RT-qPCR with the miRNAFirst Strand cDNA Synthesis and MicroRNAs qPCR Kit (Sangon Biotech, Shanghai) and the ABI plus sequence detection system (Bio-Rad, USA). The reaction conditions were 95°C for 3 minutes, followed by 40 cycles of 95°C for 10 seconds and 60°C for 30 seconds. The primer sequences for the miRNAs were designed using the online Vazyme miRNA designer tools and listed in **Supplementary Table S1**. The relative changes in expression were determined utilizing the 2^−ΔΔCt^ method (Livak and Schmittgen, 2001), with U6 serving as the internal reference gene. The PCR reactions were conducted with three biological replicates.

### 2.9 5′ RLM-RACE analysis

5′ RLM-RACE (RNA ligase-mediated rapid amplification of cDNA ends) assays were conducted to validate the predicted target genes in BM samples. The RACE assay was performed using the FirstChoice™ RLM-RACE Kit (Invitrogen, US) with slight modifications, excluding alkaline phosphatase and acid pyrophosphatase treatment of the isolated RNA. The RNA was ligated with the 5′ RACE adaptor using T4 RNA ligase. Subsequently, the ligated RNA was reverse-transcribed using random decamers and M-MLV reverse transcriptase. The generated cDNA was utilized for nested PCR, involving two rounds of amplification with primers containing the 5′ RACE outer and inner primers provided in the RLM-RACE kit, along with target gene-specific outer and inner primers designed using Primer 3 (**Supplementary Table S2**). The products from the first round of amplification served as templates for the second round. The amplified products were analyzed on a 2% agarose gel, and the expected bands were cloned into the pMD19-T vector for sequencing.

### 2.10 Statistical analysis

The data analysis in this study was performed using the statistical software SPSS version 22. Each experiment was repeated three times, and the results are presented as the mean ± standard error of the mean (SEM). All p-values were computed, and the graphs were generated using Origin Pro 2017 (Origin Lab Corporation, USA). Statistical significance was indicated by p < 0.05.

## 3 Results

### 3.1 Isolation and characterization of K-ELNs

**Figure 1:**
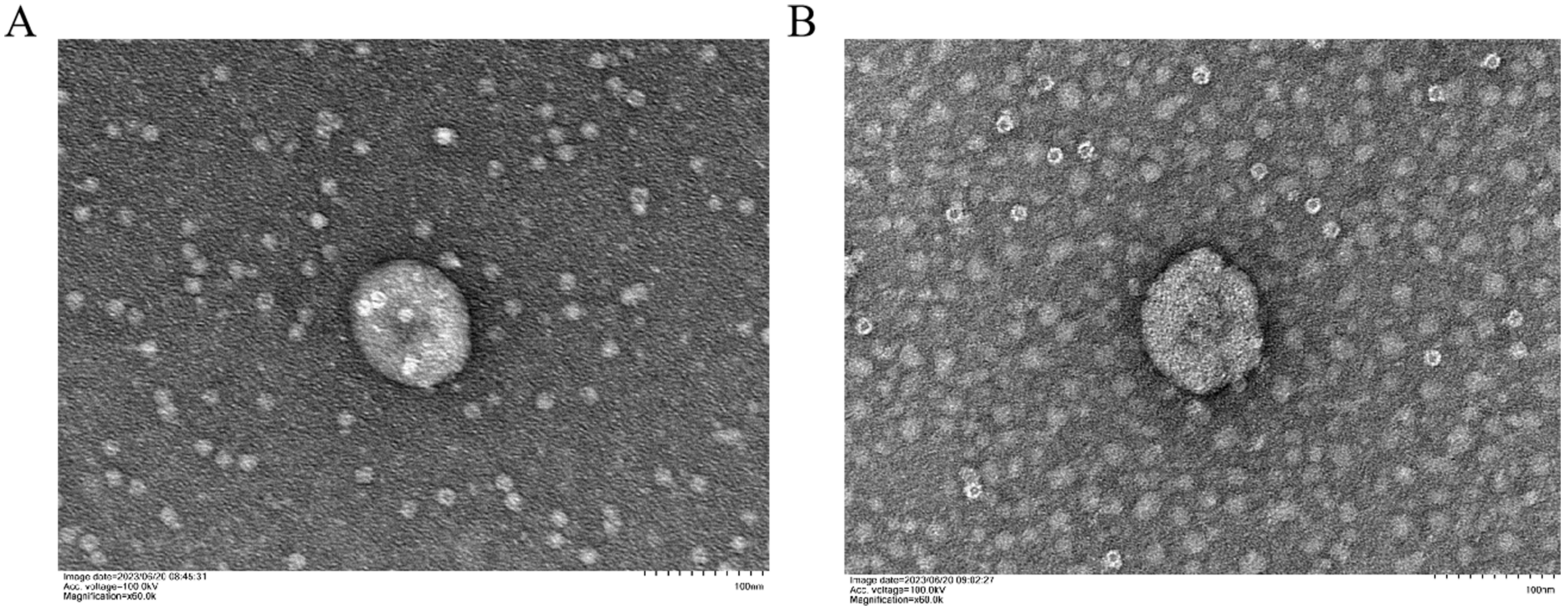
Characterization of K-ELNs. **(A)** TEM images of isolated BM_Exo. **(B)** TEM images of isolated HM_Exo. The scale is 100 nm.

Following ultracentrifugation, TEM analysis was employed to characterize the K-ELNs in four EV samples. The TEM images revealed the presence of intact vesicles isolated from both BM_Exo (**Figure 1A**) and HM_Exo (**Figure 1B**). These findings provide evidence that K-ELNs can be successfully extracted from konjac utilizing ultracentrifugation methods.

**Figure 2:**
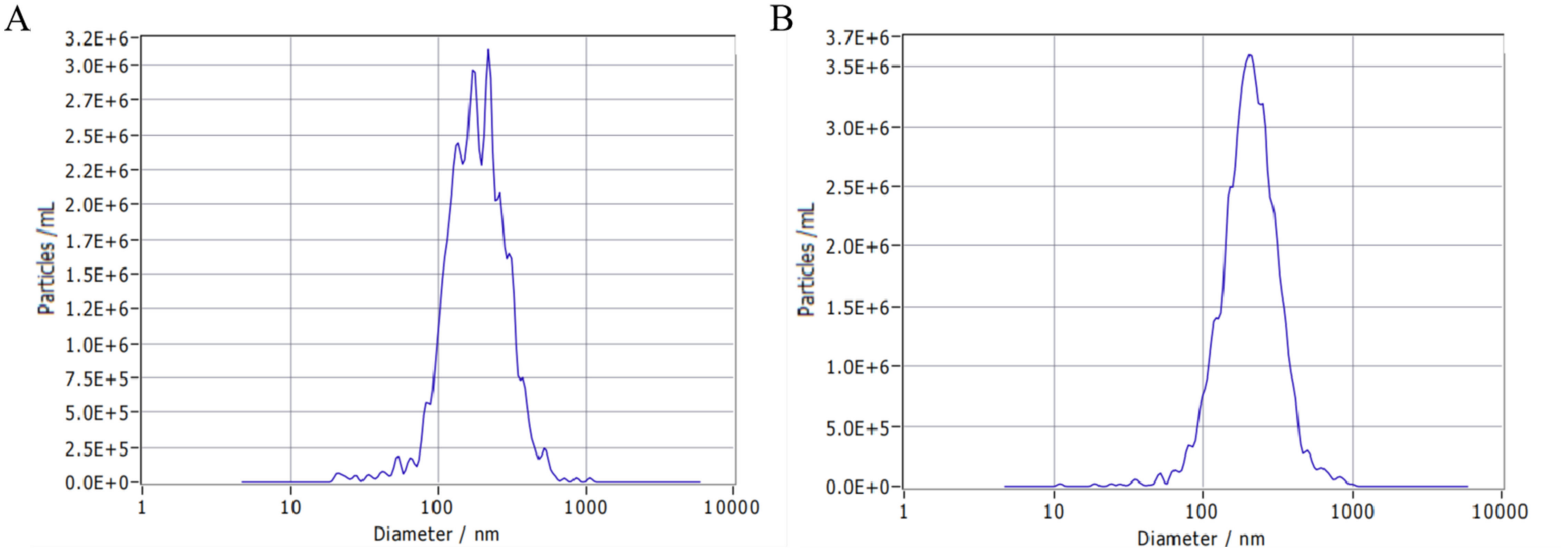
Size distribution of K-ELNs measured by NTA. **(A)** Size distribution of isolated BM_Exo. **(B)** Size distribution of isolated HM_Exo.

Subsequent to TEM analysis, NTA analysis was conducted to determine the diameter of the K-ELNs. The results indicated that the average particle size of BM_Exo and HM_Exo was 200.8 nm and 226.5 nm, respectively (**Figure 2**; **Table 1**). Furthermore, the concentration of BM_Exo and HM_Exo was measured at 8.7E+10 particles/mL and 3.9E+10 particles/mL, respectively (**Table 1**). Hence, our findings suggest that HM_Exo demonstrates a larger particle size in comparison to BM_Exo, while the concentration is notably lower than that of BM_Exo. These quantitative data further elucidate the physical characteristics and abundance of K-ELNs derived from konjac, providing valuable insights into their potential biological functions and applications.

**Table 1:** The average particle size and concentration of K-ELNs.

Sample name	Average particle size (nm)	Concentration (Particles/mL)
BM_Exo	200.8	8.7E+10
HM_Exo	226.5	3.9E+10

### 3.2 Sequencing the small RNA libraries

In order to identify miRNAs associated with ELNs and tissue in konjac, a total of 12 miRNA libraries with three biological replicates were prepared for small RNA sequencing. Strand-specific RNA sequencing of these twelve libraries generated a combined total of 293,195,720 raw paired-end reads (**Supplementary Table S3**). After removing of low-quality, adaptor, and poly-N sequences, a total of 284,884,244 clean reads were obtained for subsequent analyses (**Supplementary Table S3**).

**Figure 3:**
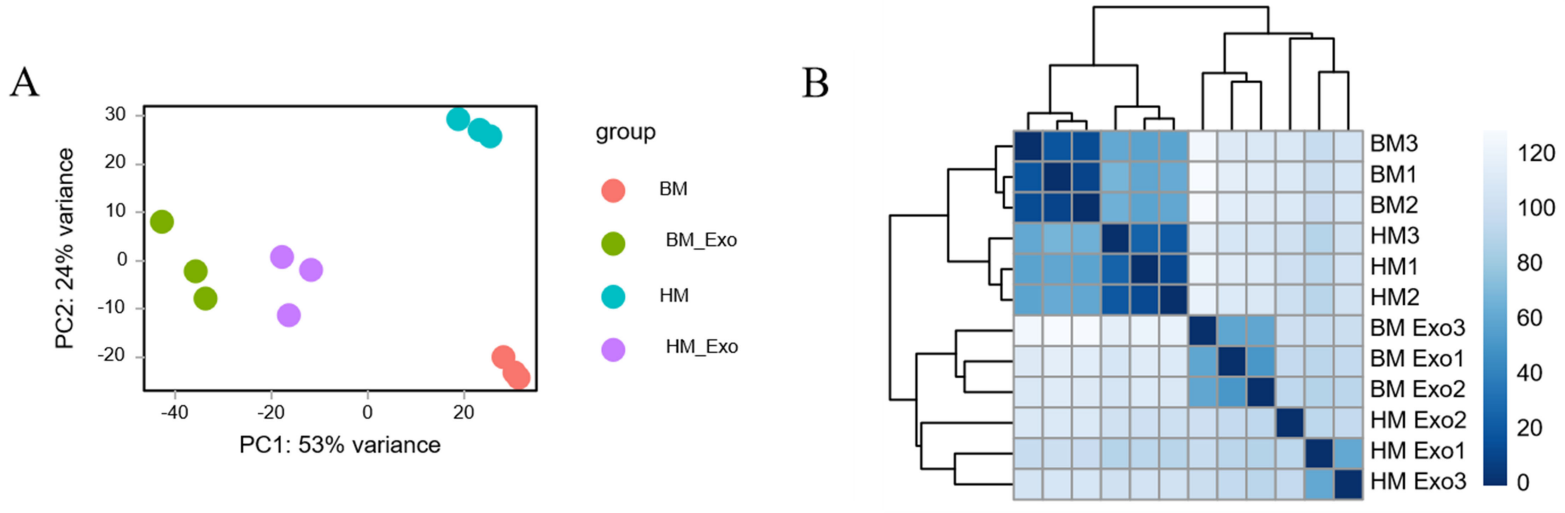
Clustering of different samples. **(A)** PCA distribution of samples based on DESeq2; **(B)** Distance analysis of samples based on DESeq2.

Principal component analysis (PCA) and distance analysis of different samples was performed to assess the homogenization of mature small RNA expression using DESeq2. The results revealed that PC1 and PC2 accounted for 53% and 24% of the variance, respectively. The samples subjected to different treatments did not cluster together, indicating effective separation. Additionally, biological replicates of the samples clustered closely together (**Figures 3A, B**).

### 3.3 Characterization of miRNAs from K-ELNs and tissues

**Figure 4:**
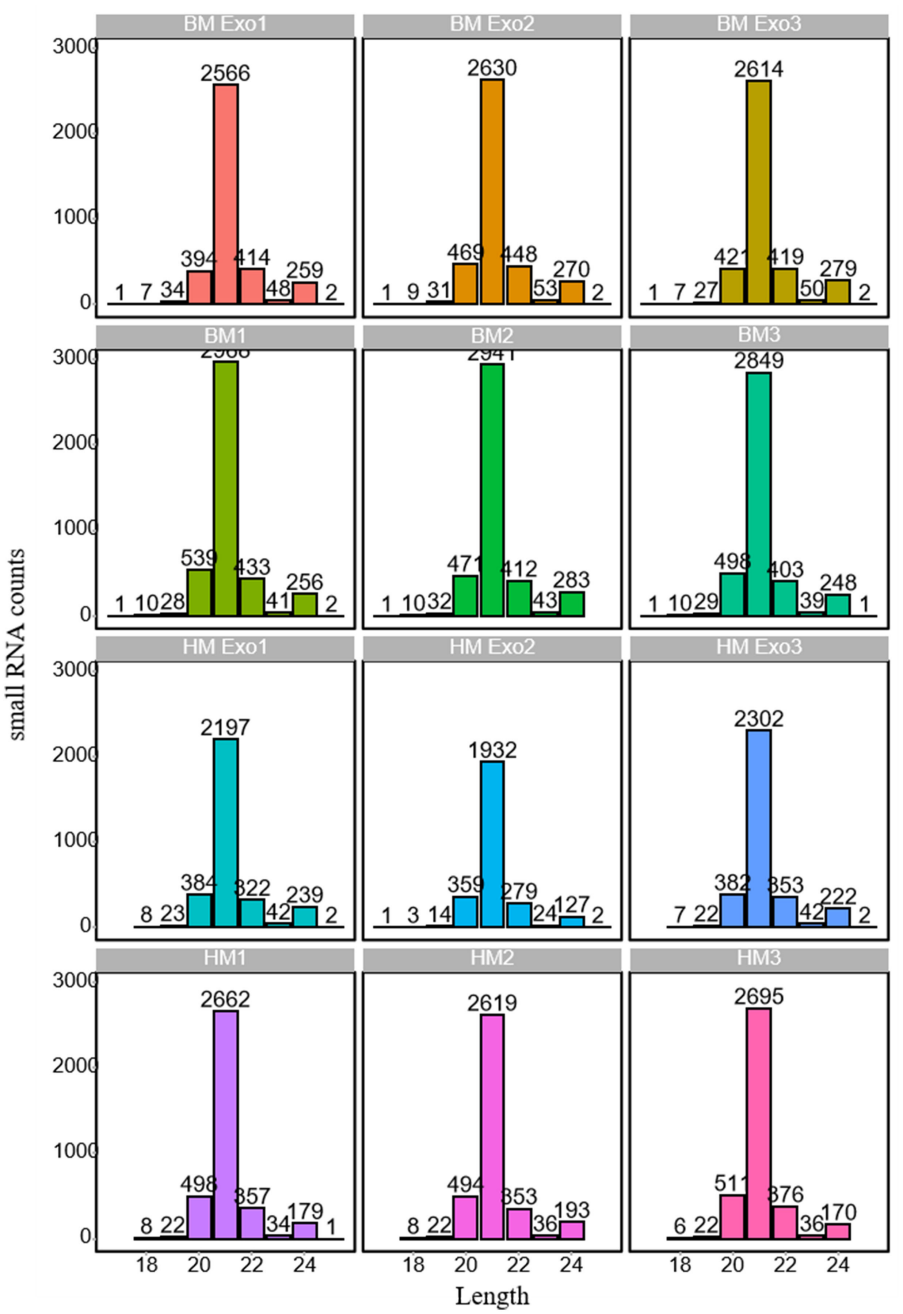
Length distribution of miRNAs in various samples. The horizontal axis represents the length of miRNAs, while the vertical axis denotes the count of small RNAs.

Following the filtration of Rfam and alignment to miRBase, the length distribution of identified miRNAs exhibiting expression levels (reads >10) was subjected to statistical analysis (**Supplementary Table S4**). The findings revealed that 21-nt miRNAs were the most abundant in both K-ELNs and tissue samples (**Figure 4**). The comparison of the screened miRNAs with all mature miRNA sequences in the miRBase database provides insights into the degree of similarity between the identified small RNA sequences and those of other species. The results showed that the species with the highest miRNA similarity to those in konjac were *Glycine max* (L.) Merr., *Oryza sative* L., *Medicago truncatula* Gaertn, and *Picea abies* (L.) H. Karst (**Supplementary Figure S1**).

By counting the number of miRNAs in all samples, the top 20 most abundant miRNA families were screened out. The results indicated that the most abundant miRNA families were miR156a, miR166b, miR166a, and miR156b in both K-ELNs and tissue samples (**Supplementary Figure S2**). Nevertheless, the miRNA families including miR408, miR166e, miR156d, and miR169a exhibited a higher abundance in BM_Exo compared to BM, while the miRNA families miR393a, miR319b, and miR166f showed a higher abundance in HM_Exo compared to HM. Furthermore, an analysis of the top 20 miRNA families with the highest expression levels in each sample was conducted. The findings revealed that the families of miR535e, miR535c, miR535b, miR535-5p, miR535a-5p, miR535b-5p, miR535a, miR535, and miR535d exhibited high expression levels in both BM and BM_Exo samples. In contrast, the expression patterns of miRNAs in HM_Exo differed from those in HM (**Supplementary Figure S3**).

### 3.4 Differential expression analysis of miRNA between K-ELNs and tissue

**Figure 5:**
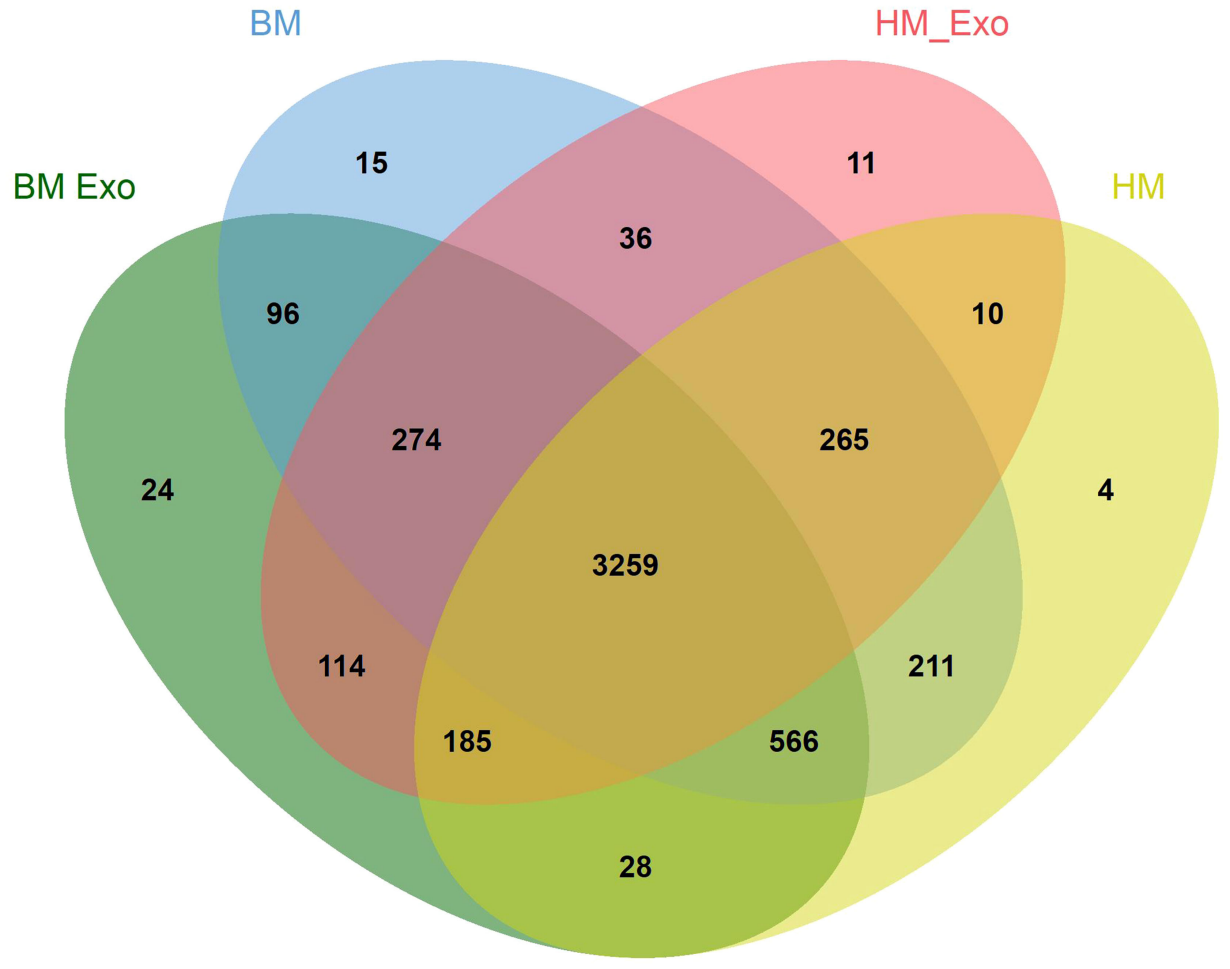
Venn plots depicting the distribution of miRNAs across various samples.

Through an analysis of all miRNAs in comparison to miRBase, a venn diagram was constructed to visually represent the distribution of all identified miRNAs across the samples. The findings revealed the presence of a total of 3259 miRNAs in all samples. Additionally, there were 15, 24, 4, and 11 miRNAs unique to the samples of BM, BM_Exo, HM, and HM_Exo, respectively (**Figure 5**).

**Figure 6:**
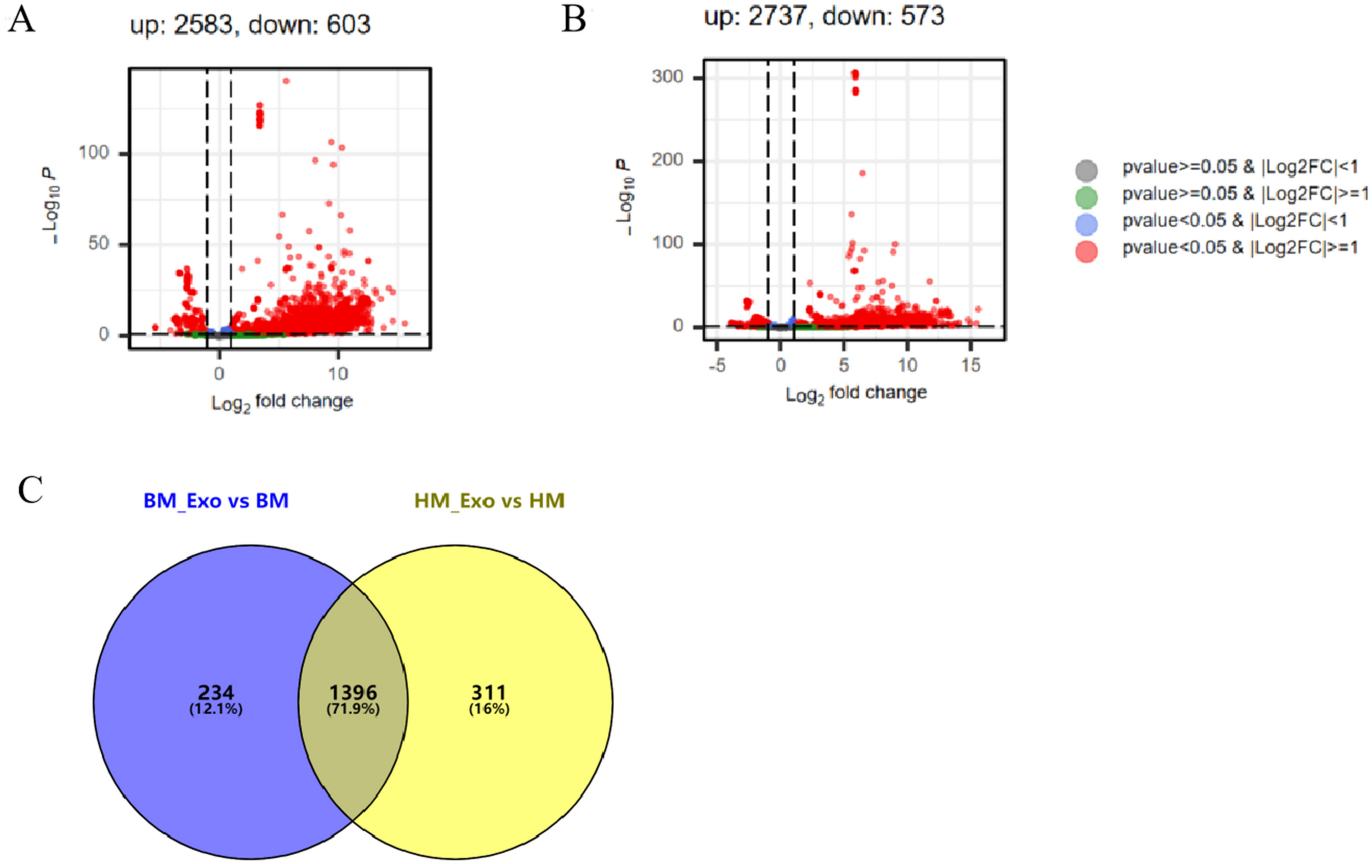
The statistics of DEMs between K-ELNs and tissue. **(A)** Volcano plot of the different expression patterns between BM_Exo and BM. **(B)** Volcano plot of the different expression patterns between HM_Exo and HM. **(C)** Venn diagram of DEMs in two different comparison combinations.

Upon comparing the DEMs identified in K-ELNs and tissues, a significant disparity was observed. In the comparison between BM_Exo and BM, 2583 miRNAs were identified as up-regulated, while 603 miRNAs were down-regulated (**Figure 6A**). Similarly, in the comparison between HM_Exo and HM, 2737 miRNAs were found to be up-regulated, with 573 miRNAs down-regulated (**Figure 6B**). The analysis of differential miRNAs in the two distinct comparative combinations unveiled that 1,396 miRNAs were common between them. Among these, 234 miRNAs were exclusive to the BM_Exo *vs* BM comparison, while 311 miRNAs were specific to the HM_Exo *vs* HM comparison (**Figure 6C**).

**Figure 7:**
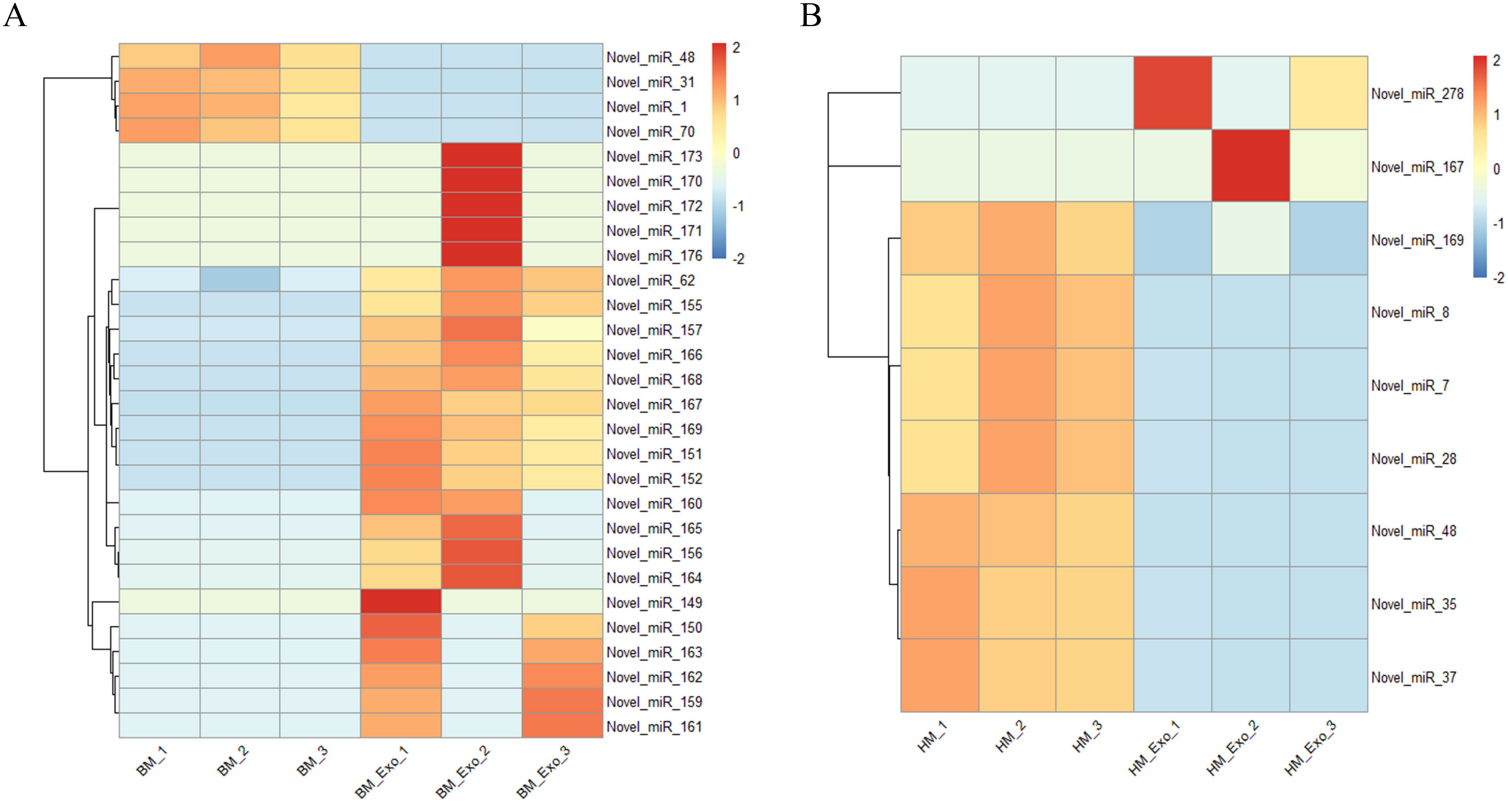
Differential expression of novel miRNAs in K-ELNs and tissues. **(A)** Heatmap of the different expression novel miRNAs between BM_Exo and BM. **(B)** Heatmap of the different expression novel miRNAs between HM_Exo and HM.

In addition, the expression of the novel miRNAs in different samples was also analyzed, and the results showed that 28 novel miRNAs, including 24 up-regulated miRNAs and 4 down-regulated miRNAs, were differential expression in the comparison between BM_Exo and BM (**Figure 7A**). However, 2 up-regulated and 7 down-regulated novel miRNAs were differential expression in the comparison between HM_Exo and HM (**Figure 7B**).

### 3.5 Functional enrichment analysis of target genes in konjac

**Figure 8:**
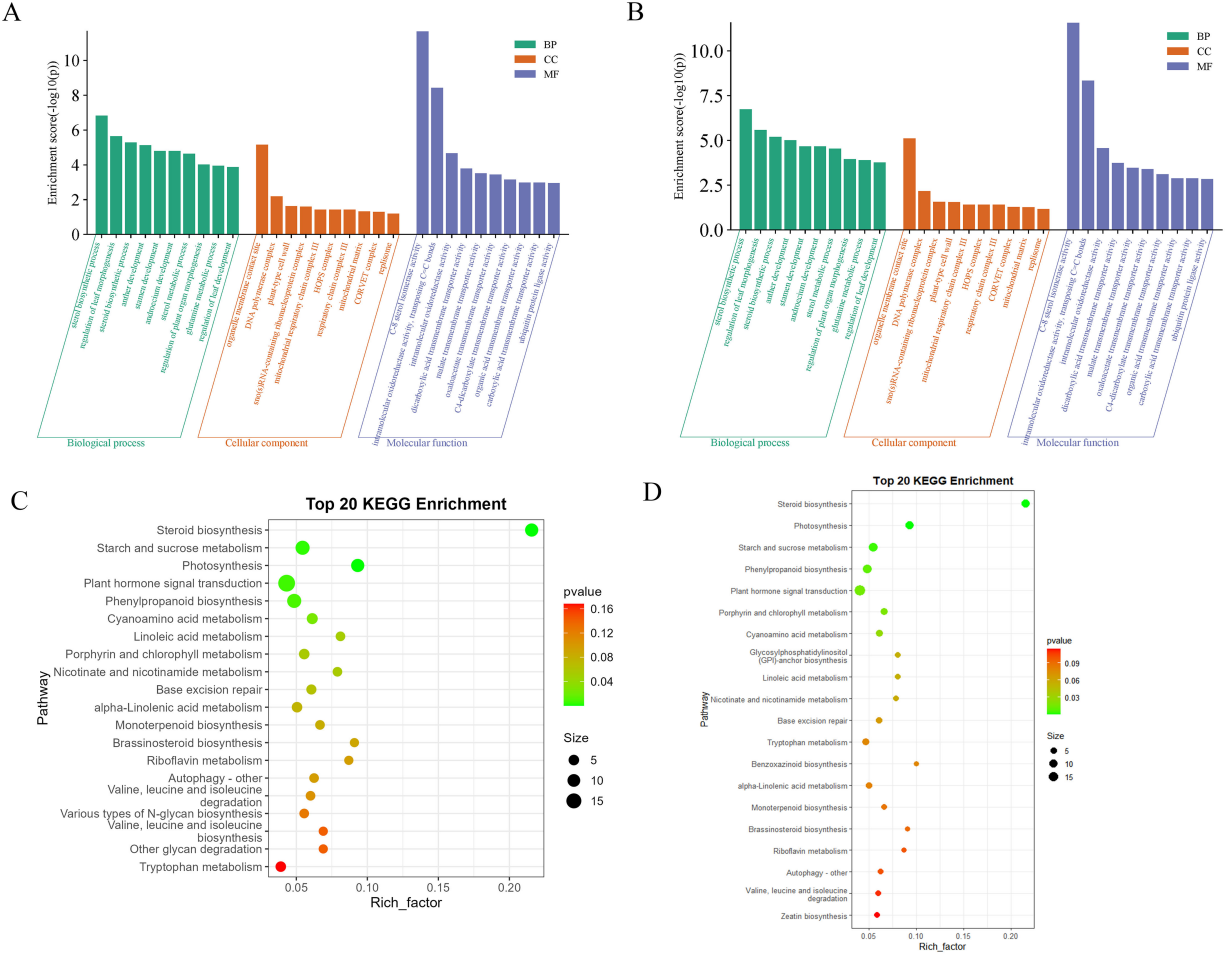
Functional annotation of the predicted target genes of the DEMs in konjac. **(A)** GO classification of the target genes of BM_Exo *vs* BM. **(B)** GO classification of the target genes of HM_Exo *vs* HM. **(C)** KEGG enrichment analysis of the target genes of BM_Exo *vs* BM. **(D)** KEGG enrichment analysis of the target genes of HM_Exo *vs* HM.

In order to investigate the functional implications of these DEMs in konjac, target genes were predicted using the miRanda software. Subsequently, after removing redundancy, a total of 1102 and 1119 target genes were identified in the BM_Exo and HM_Exo samples, respectively. These target genes were further subjected to GO and KEGG annotation analyses for functional characterization. The GO annotation of the target genes revealed that miRNAs from K-ELNs potentially regulate konjac genes involved in various functions (**Figures 8A, B**). Interestingly, the pathway of GO functional enrichment in K-ELNs from both BM_Exo and HM_Exo samples was found to be similar. In terms of biological processes, the predicted targets were predominantly associated with the sterol biosynthetic process and regulation of leaf morphogenesis. Within the cellular component category, these targets were primarily linked to organelle membrane contact sites and DNA polymerase complexes. Regarding molecular functions, the targets were primarily associated with C-8 sterol isomerase activity and intramolecular oxidoreductase activity.

The KEGG functional enrichment pathway analysis of K-ELNs from both BM_Exo and HM_Exo samples revealed a similar pattern (**Figures 8C, D**). The top 20 KEGG pathways associated with the target genes exhibited significant enrichment in pathways such as plant hormone signal transduction, steroid biosynthesis, starch and sucrose metabolism, photosynthesis, and phenylpropanoid biosynthesis.

### 3.6 Functional enrichment analysis of target genes in human

**Figure 9:**
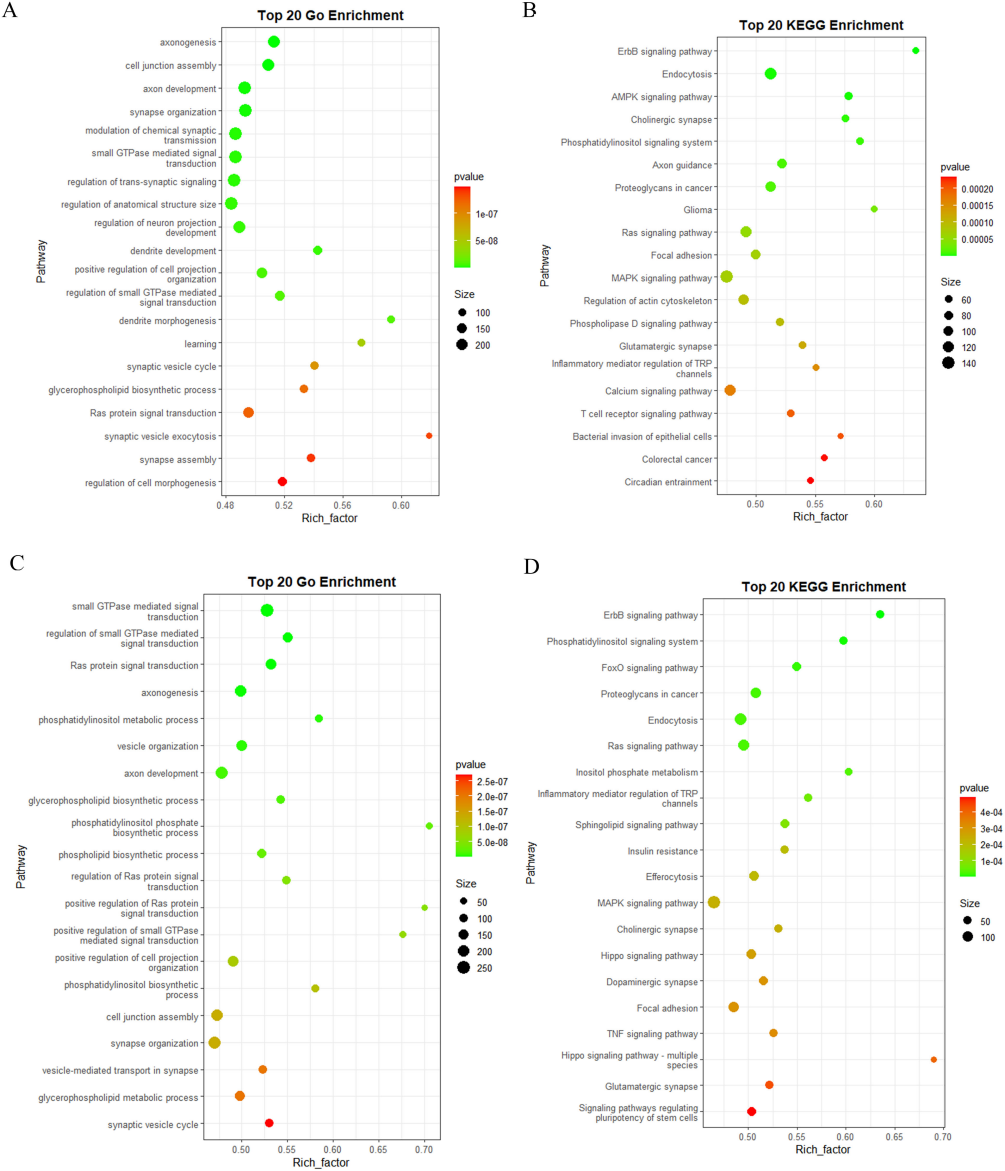
Functional annotation of the predicted target genes of the DEMs in human. **(A)** GO classification of the target genes of BM_Exo *vs* BM. **(B)** KEGG enrichment analysis of the target genes of BM_Exo *vs* BM. **(C)** GO classification of the target genes of HM_Exo *vs* HM. **(D)** KEGG enrichment analysis of the target genes of HM_Exo *vs* HM.

To elucidate the target genes regulated by DEMs and their implications for human health, GO and KEGG enrichment analyses were performed on the annotated target genes. Given the low expression levels of certain miRNAs, we focused on the top 300 DEMs for target gene prediction and enrichment analysis within the human genome. The GO enrichment analysis results revealed that the target genes of DEMs in BM_Exo were primarily associated with small GTPase-mediated signal transduction, cell junction assembly, synapse organization, ras protein signal transduction, and axonogenesis (**Figure 9A**). Furthermore, the KEGG enrichment analysis indicated that the target genes of DEMs in BM_Exo were predominantly linked to the MAPK signaling pathway, calcium signaling pathway, endocytosis, proteoglycans in cancer, and ras signaling pathway (**Figure 9B**).

Similar yet subtly different outcomes were observed in HM. The GO enrichment analysis results showed that the target genes of DEMs in HM_Exo were mainly related to small GTPase-mediated signal transduction, axonogenesis, synapse organization, and cell junction assembly (**Figure 9C**). Additionally, the KEGG enrichment analysis revealed that the target genes of DEMs in HM_Exo were primarily associated with the MAPK signaling pathway, endocytosis, ras signaling pathway, signaling pathways regulating pluripotency of stem cells, and hippo signaling pathway (**Figure 9D**).

**Figure 10:**
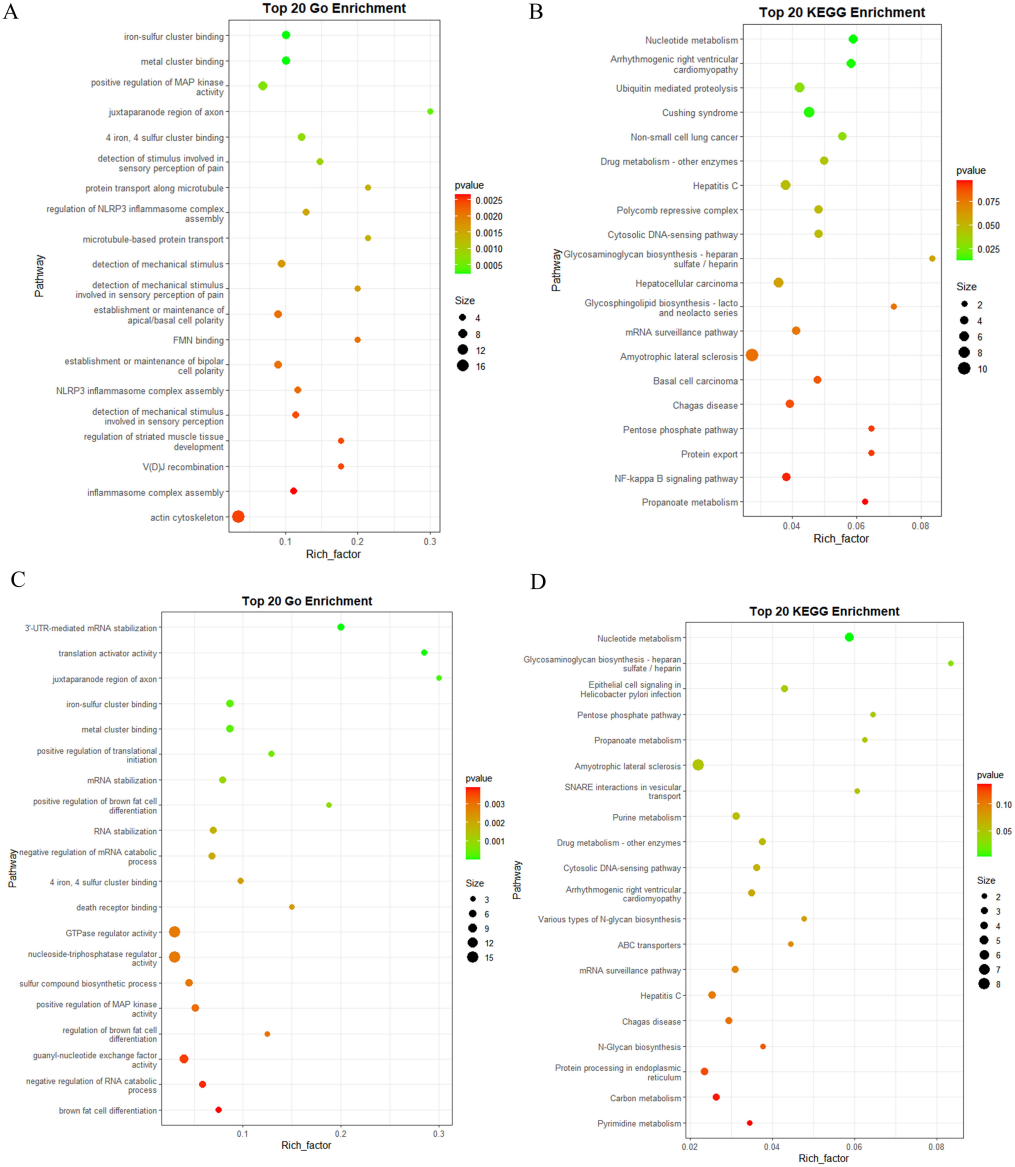
Functional annotation of the predicted target genes of the differential expression novel miRNA in human. **(A)** GO classification of the target genes of BM_Exo *vs* BM. **(B)** KEGG enrichment analysis of the target genes of BM_Exo *vs* BM. **(C)** GO classification of the target genes of HM_Exo *vs* HM. **(D)** KEGG enrichment analysis of the target genes of HM_Exo *vs* HM.

Novel miRNAs also play a crucial role in elucidating the functions of K-ELNs. The results of the GO enrichment analysis revealed that the target genes of novel miRNAs in BM_Exo were primarily associated with the actin cytoskeleton, positive regulation of MAP kinase activity, NLRP3 inflammasome complex assembly, and metal cluster binding (**Figure 10A**). Additionally, the KEGG enrichment analysis indicated that the target genes of novel miRNAs in BM_Exo were predominantly linked to amyotrophic lateral sclerosis, hepatocellular carcinoma, the NF-kappa B signaling pathway, and Cushing syndrome (**Figure 10B**).

The results of the GO enrichment analysis revealed that the target genes of novel miRNAs in HM_Exo were primarily associated with the GTPase regulator activity, nucleoside-triphosphatase regulator activity, and guanyl-nucleotide exchange factor activity (**Figure 10C**). Additionally, the KEGG enrichment analysis indicated that the target genes of novel miRNAs in BM_Exo were predominantly linked to amyotrophic lateral sclerosis, Hepatitis C, Chagas disease, and epithelial cell signaling in helicobacter pylon infection (**Figure 10D**).

### 3.7 MiRNA expression validation by RT-qPCR

**Figure 11:**
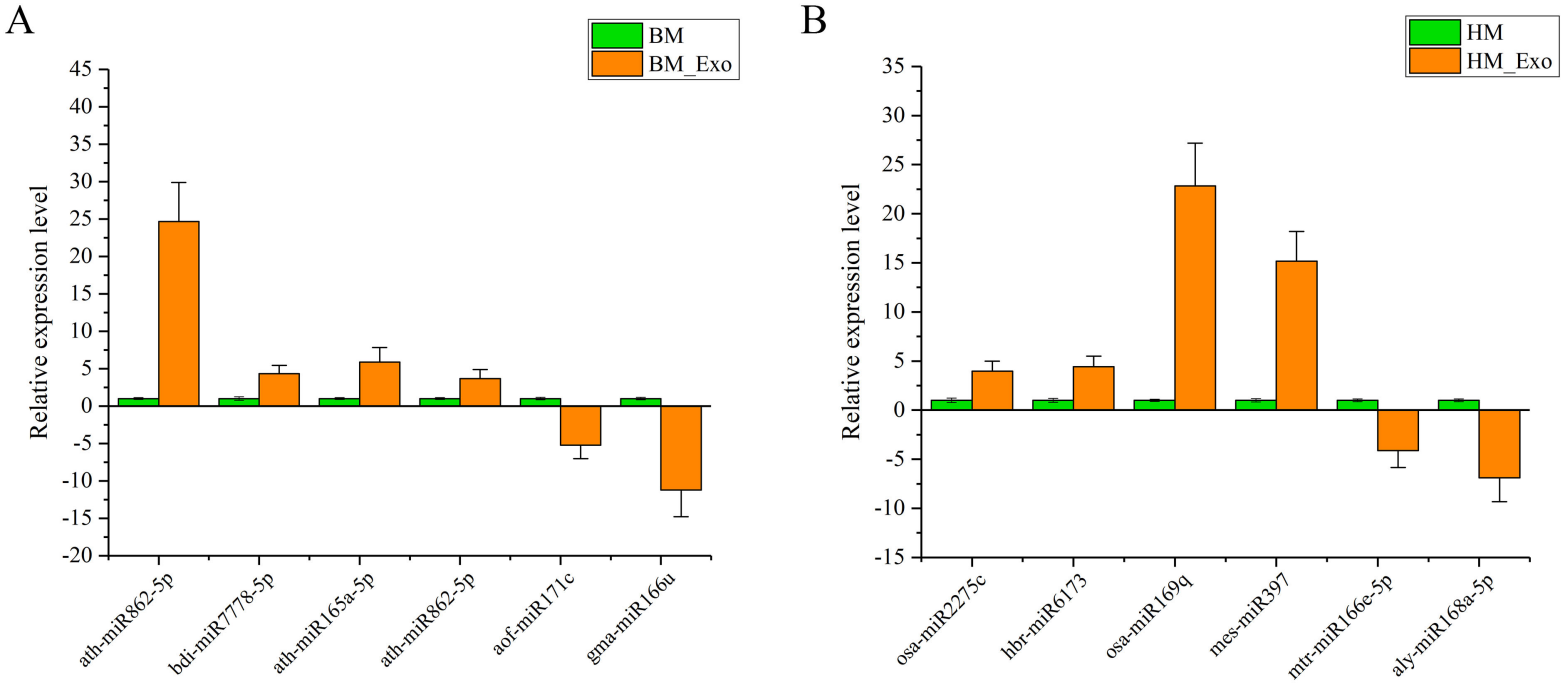
Expression profiles of selected miRNAs in K-ELNs and Tissue. **(A)** Relative expression level of six miRNAs between BM_Exo and BM. **(B)** Relative expression level of six miRNAs between BM_Exo and BM. Data are the means of three replicates (± SD).

Due to the low expression levels of numerous miRNAs, a screening process was conducted to identify miRNAs with detectable expression levels, ensuring the feasibility of quantitative experiments across various samples. Consequently, four up-regulated miRNAs and two down-regulated miRNA were selected from both the BM_Exo and HM_Exo samples for validation. These miRNAs were subjected to RT-qPCR analysis to confirm the findings from RNA-seq (**Figure 11**). The results demonstrated a high consistency between the RT-qPCR expression levels and the RNA-seq data, indicating the reliability of the RNA-seq results and their suitability for further analysis.

### 3.8 Validation of the predicted target gene by 5’RLM-RACE

**Figure 12:**
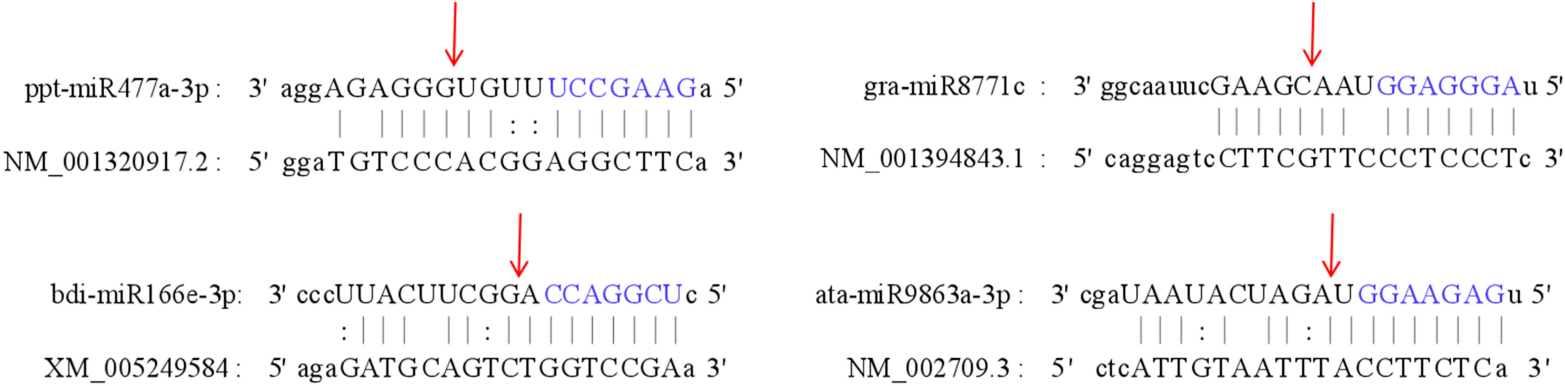
Validation of the interaction between four miRNAs and their target genes using 5’RLM-RACE in BM. The cleavage sites are indicated by the red arrows, while the seed sequence of the miRNA is highlighted in blue.

To validate the accuracy of the predicted miRNA targets, 5’ RLM-RACE was employed to confirm the interactions between DEMs and their predicted target genes (**Figure 12**). While the sequences of the nested PCR products did not reveal the cleavage sites for many mRNAs, four pairs were successfully detected. The selected target genes were associated with the human disease resistance pathway, including the cholinergic receptor muscarinic 5 (CHRM5, NM_001320917.2), Wnt family member 7B (WNT7B, NM_058238.3), adenylate cyclase 1 (ADCY1, XM_005249584, and phosphatase 1 catalytic subunit beta (PPP1CB, NM_002709.3). The results revealed cleavage sites between the 12th and 13th nucleotides of ppt-miR477a-3p and the CHRM5 transcript, the 11th and 12th nucleotides of gra-miR8771c and the WNT7B transcript, the 9th and 10th nucleotides of bdi-miR166e-3p and the ADCY1 transcript, and the 9th and 10th nucleotides of ata-miR9863a-3p and the PPP1CB transcript.

## 4 Discussion

Konjac, primarily composed of glucomannan, has extensive applications in both the food and pharmaceutical fields (Devaraj et al., 2019). In recent years, with the increasing emphasis on healthy eating, Konjac has garnered significant attention due to its unique nutritional value and numerous health benefits. In the realm of human health, Konjac is recognized for its ability to prevent atherosclerosis, treat cardiovascular and cerebrovascular diseases, lower cholesterol levels, enhance immunity, prevent tumors and cancer, as well as reduce blood sugar and lipid levels (Xu et al., 2022; Zhang and Rhim, 2022). The predominant method currently for extracting edible plant-derived ELNs is through the use of ultracentrifugation, which has demonstrated significant efficacy and promising outcomes (Liu et al., 2022; Hwang et al., 2023). In our study, the findings related to the isolation, characterization, and sequencing of K-ELNs provide novel insights into their biophysical properties and potential biological functions. The successful extraction and characterization of K-ELNs using ultracentrifugation, followed by analysis through TEM and NTA, underscore the feasibility of isolating intact vesicles with high purity and concentration. Our observations are consistent with previous studies that have demonstrated the effectiveness of ultracentrifugation in isolating extracellular vesicles from various biological matrices (Tian et al., 2019; Liangsupree et al., 2021). The observed differences in size distribution and concentration of K-ELNs between BM_Exo and HM_Exo may reflect underlying biological variations.

The miRNAs encapsulated within these plant-derived ELNs have been implicated in a variety of physiological and pathological processes, underscoring their potential as therapeutic agents and diagnostic biomarkers (Mu et al., 2023). Edible plant miRNAs are characterized by 2’-O-methylated modifications at their 3’ ends and a high GC content. Additionally, their exosomal packaging confers high stability, enabling them to withstand the harsh environments of the digestive and circulatory systems (Samad et al., 2021; Xu et al., 2023). Some recent studies have demonstrated that plant-derived miRNAs, particularly those obtained through dietary intake, have the capability to enter the host circulation and interact with target cells, thereby modulating gene expression (Lian et al., 2022). However, due to the vast diversity of plant species, the precise functions of these miRNAs remain largely elusive in current research. In our study, the sequencing of small RNA libraries from K-ELNs revealed a rich diversity of miRNAs, with a notable abundance of 21-nt miRNAs. This finding is consistent with previous studies suggesting that miRNAs of this size class are predominant in extracellular vesicles, playing pivotal roles in intercellular communication and the regulation of gene expression (Takahashi et al., 2017). Moreover, the miR166 and miR155 families were found to be the most abundant, aligning with the findings reported for blueberry-derived ELNs (Leng et al., 2024). Through the analysis of miRNAs across all samples, it was observed that 24 miRNAs were exclusively expressed in BM_Exo samples, while 11 miRNAs were unique to HM_Exo samples. These uniquely expressed miRNAs may serve as key determinants of their functional distinctions.

Emerging evidence from clinical and preclinical studies indicates that plant-derived EVs offer various advantages over traditional synthetic carriers, thereby paving the way for innovative advancements in drug delivery systems (Nemati et al., 2022; Feng et al., 2023). The functional enrichment analysis of target genes predicted for the DEMs in Konjac provides insights into the biological processes and pathways potentially regulated by these miRNAs. The enrichment in pathways related to plant hormone signal transduction, sterol biosynthesis, and photosynthesis not only corroborates the regulatory roles of miRNAs in plant physiology but also underscores the potential of K-ELNs in influencing plant growth, development, and stress responses. Similarly, the functional enrichment analysis of target genes in the human genome reveals the therapeutic potential of K-ELNs and their miRNAs in modulating pathways associated with diseases, including cancer and neurodegenerative disorders. Notability, despite the distinctions between BM and HM as different konjac species with varying traits, the functional variances in exosomal microRNA target genes are not substantial, suggesting that both konjac species serve similar functions for both human health and plant physiology.

It is widely recognized that edible plant-derived ELNs exert a significant impact on mammalian cell homeostasis within the digestive system (Yin et al., 2022). The digestive system has the capacity to absorb plant miRNAs and release them into the circulatory system. Subsequently, exogenous plant microRNAs can be transported to target cells in various organs, where they regulate the functions of recipient cells (Jia et al., 2021). Previous studies have demonstrated that ELNs derived from four edible plants, namely grape, grapefruit, ginger, and carrot, were internalized by intestinal macrophages and stem cells (Mu et al., 2014). For instance, a previous study identified miR-156e, miR-162, and miR-319d from blueberry-derived ELNs as potential regulators associated with the down-regulation of prostaglandin I2 synthase (PTGIS), mitogen-activated protein kinase 14 (MAPK14), and phosphodiesterase 7A (PDE7A), influencing the response to tumor necrosis factor-α (TNF-α) (De Robertis et al., 2020). Another study reported that plant miR159, which is enriched in women’s serum extracellular vesicles, exhibited an inverse association with the progression of breast cancers and morbidity (Gong et al., 2019). Additionally, a separate study revealed that gma-miR-3600, a highly enriched miRNA in ginger-derived ELNs, targeted the SARS-CoV-2 genome (Kalarikkal and Sundaram, 2021). Similarly, plant miR2911 found in honeysuckle soup has been shown to inhibit the replication of SARS-CoV-2 and expedite the recovery of infected patients (Zhou et al., 2020). Plant miR168a binds to the LDLRAP1 (LDL receptor adapter protein 1) gene, leading to decreased LDLRAP1 levels in liver cells and subsequently reducing the clearance of LDL cholesterol (Zhang et al., 2011). Consistently, these functional miRNAs were also identified in K-ELNs, such as miR168, miR165, miR159, miR156, and miR319. Moreover, a significant portion of the DEMs were found to target human genes involved in various immune and metabolic-related biological pathways (**Supplementary Table S5**).

While our study provides valuable insights into the potential roles of K-ELNs and their miRNAs, it is important to acknowledge the limitations of our approach. The use of the *A. konjac* genome as a reference for analyzing *A. albus* sequencing data may have introduced biases in our results. Future studies should aim to sequence and annotate the *A. albus* genome to provide a more accurate reference for comparative genomic analyses. Additionally, further *in vivo* and *in vitro* studies are required to validate our findings and investigate the specific roles of miRNAs in K-ELNs in relation to human health. These studies will be crucial in elucidating the full potential of K-ELNs as therapeutic agents and their impact on human physiology.

## 5 Conclusion

Overall, this study provides a comprehensive analysis of the physical and molecular characteristics of K-ELNs, establishing a robust foundation for future investigations into their biological functions and applications. High-throughput sequencing was employed to analyze the miRNA profiles in konjac and K-ELNs, with the DEMs in K-ELNs predicted to play a role in regulating pathways associated with human diseases. The findings underscore the intricate mechanisms of miRNA-mediated gene regulation and signaling, providing avenues for further exploration into the functional significance of K-ELNs and their miRNA content in biological processes and disease pathways.

## Data availability statement

The original contributions presented in the study are included in the article/**Supplementary Material**. Further inquiries can be directed to the corresponding author.

## Author contributions

CS: Writing – review & editing, Writing – original draft, Supervision, Project administration, Funding acquisition, Formal analysis, Data curation. XL: Writing – review & editing, Visualization, Software, Investigation, Data curation. JQ: Writing – review & editing, Validation, Software, Resources, Methodology, Conceptualization. LD: Writing – review & editing, Visualization, Validation, Methodology, Formal Analysis.

## Supplementary material

Supplementary Table 1 List of the miRNA primers used for RT-qPCR.

Supplementary Table 2 List of the PCR primers used for 5’RLM-RACE.

Supplementary Table 3 Summary of sequencing data from 12 libraries.

Supplementary Table 4 The normalized raw counts of miRNAs detected in the sequencing libraries.

Supplementary Table 5 The Top20 expressed miRNAs and their targets in the human genome.
