# Supplementary figures and images for: Characterization of miRNA profiling in konjac-derived exosome-like nanoparticles and elucidation of their multifaceted roles in human health
Source: Front Plant Sci. 2024 Aug 8;15:1444683. doi: 10.3389/fpls.2024.1444683 (PMC11338808; doi:10.3389/fpls.2024.1444683)

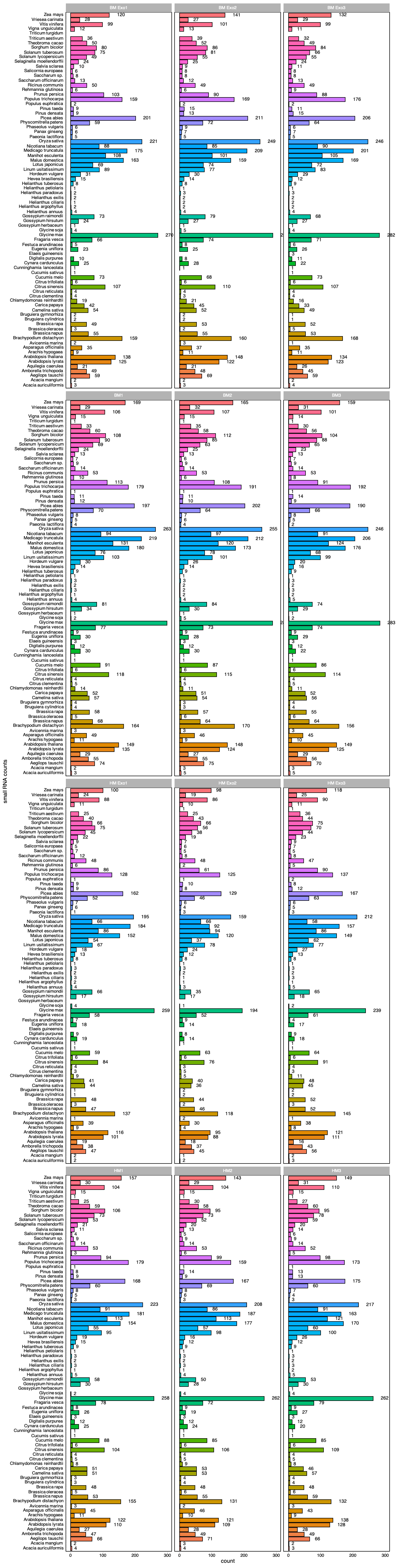

Supplement: Supplementary file 2 [file Image_1.tif]

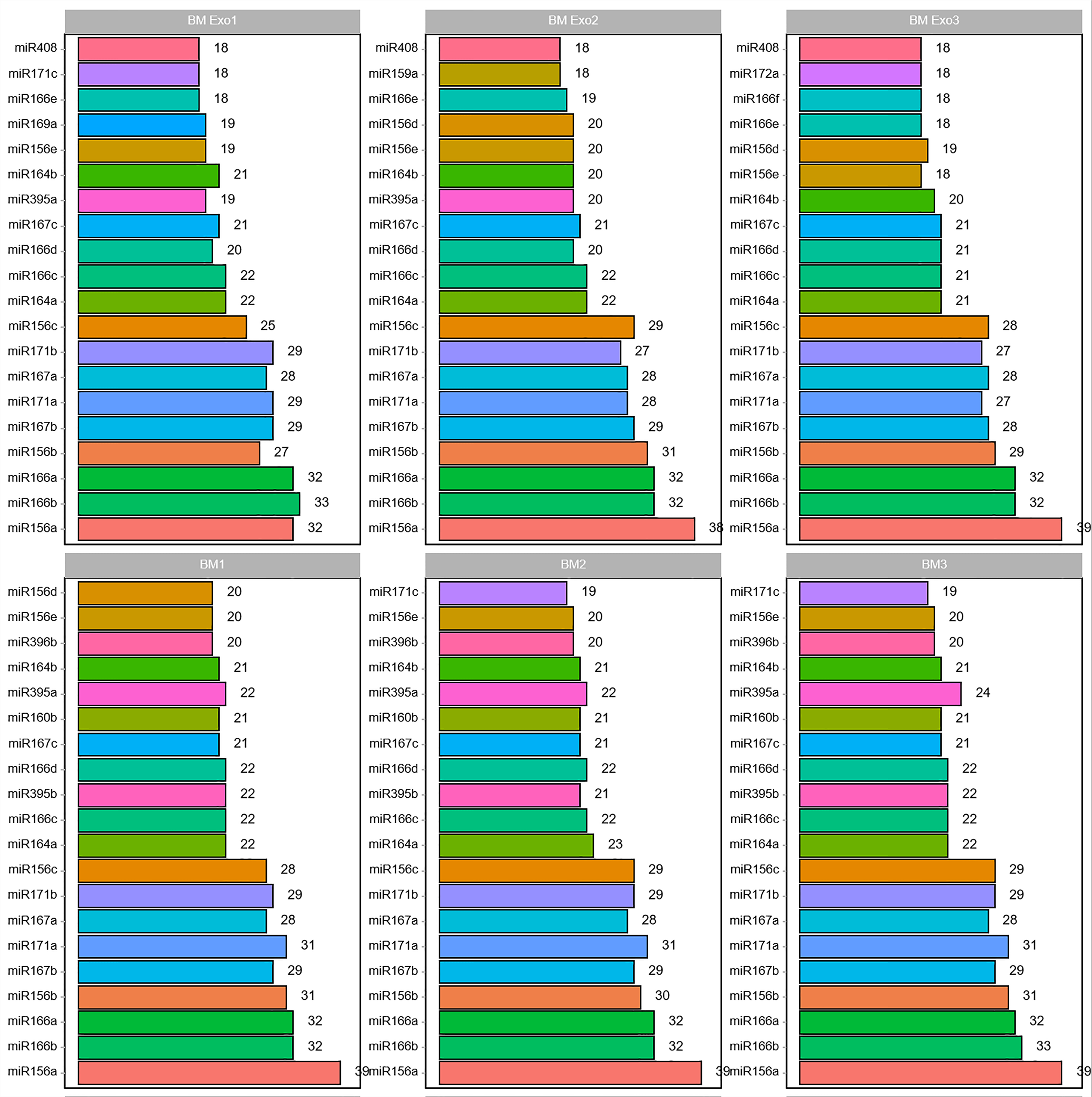

Supplement: Supplementary file 3 [file Image_2.tif]

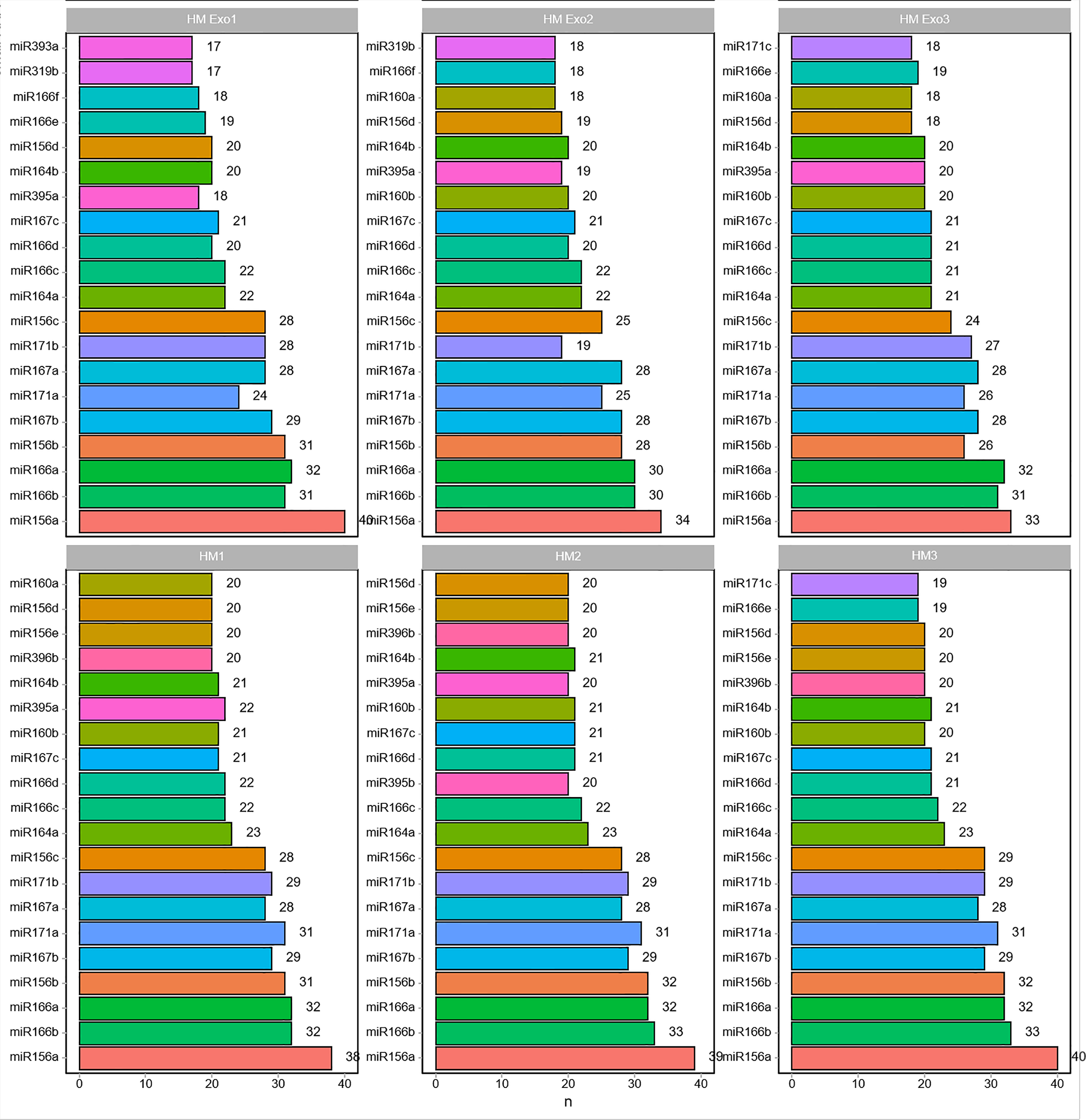

Supplement: Supplementary file 4 [file Image_3.tif]

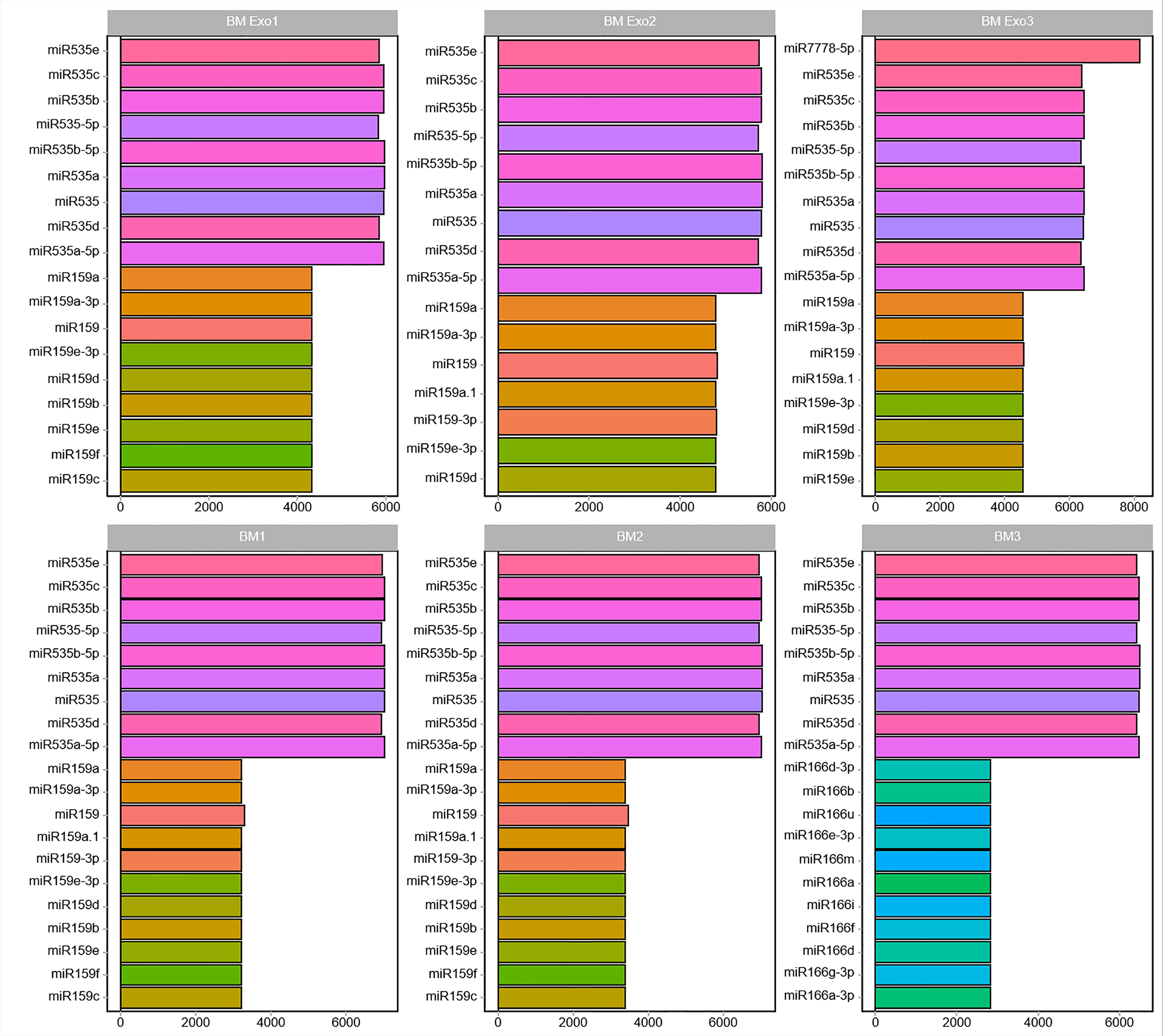

Supplement: Supplementary file 5 [file Image_4.tif]

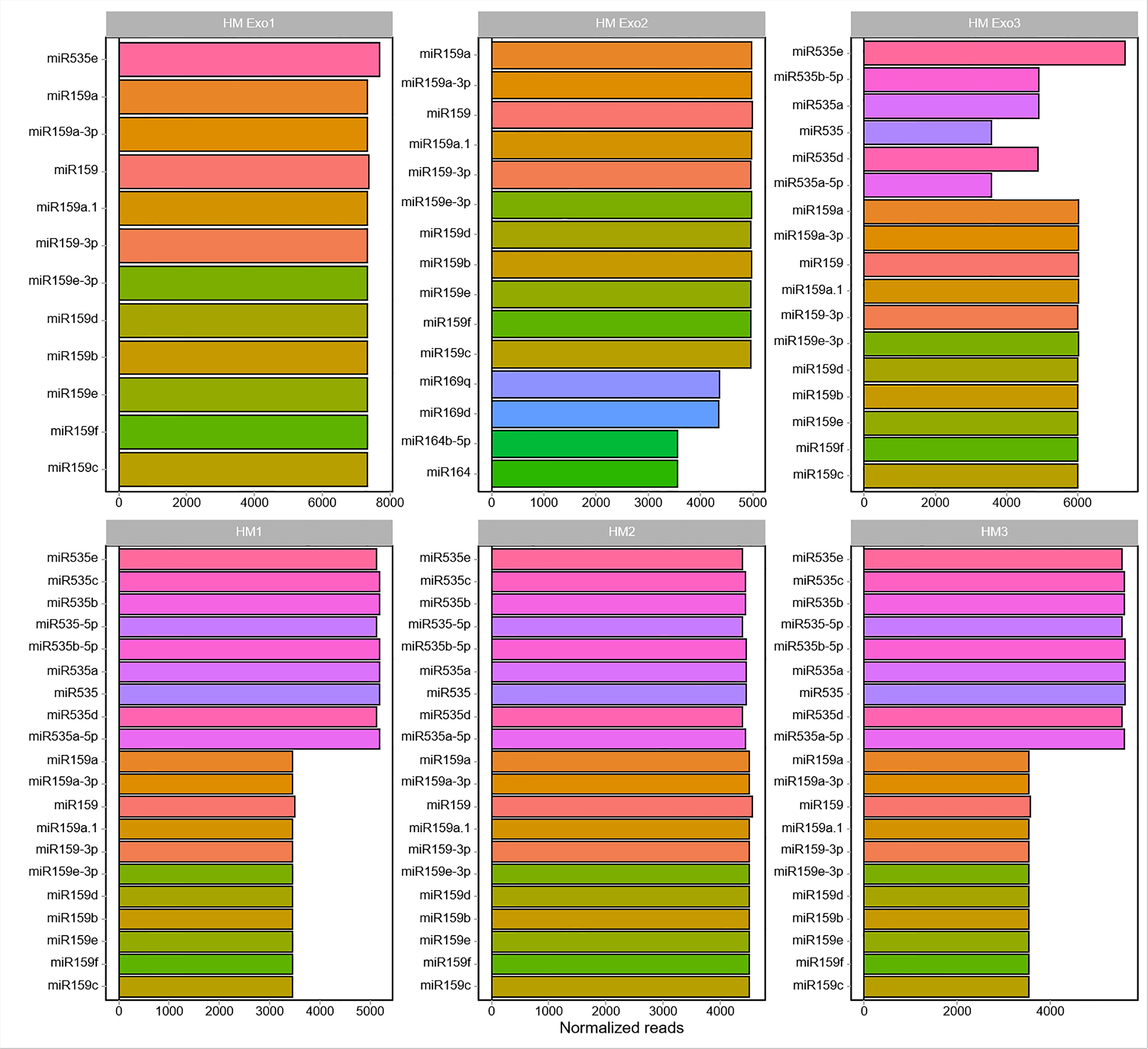

Supplement: Supplementary file 6 [file Image_5.tif]
